# Some Poor-Law Accounts

**Published:** 1903-04-25

**Authors:** 


### SOME POOR-LAW ACCOUNTS.

If Poor-law accounts were kept on a uniform system, as so many hospital accounts now are, they would probably be of more use to the officials concerned with them, as affording a clear basis of comparison. On the other hand they would lose an interest which they now possess, in showing what are the points which different boards consider of sufficient importance to be stated in detail. We have before us the accounts of three different parishes, those of Glasgow, of New Monkland (an industrial parish in Lanarkshire), and of the little town and parish of St. Peter Port, in Guernsey. The conditions in these parishes are sufficiently different to make their statistics of pauperism reading which one would peruse with some curiosity, but our present purpose is rather to point out the things which are mentioned in one report and not in the others.

The Glasgow report is as full and interesting as one would expect from a man who takes such a real and humane interest in his work as inspector of poor as Mr. Motion. One notes with regret that there is a slight increase of pauperism in 1902 as compared with 1901. This may be due either to a slackness of the ship-building industry—though that would perhaps tell more heavily in Govan than in Glasgow itself—or it may be only the natural reaction after the prosperity which was brought by the successful Exhibition of 1901. The general summary shows how many of the applications for relief are new, and how many old, and since November 1901, a separate list has been kept of "ins-and-outs," which therefore no longer go to swell the total of permanent pauperism. A further analysis shows the number of times that the same applicant appears to ask for relief. This is tested by the admissions to the poorhouses belonging to the parish. Thus while 2,881 persons entered the Barnhill poorhouse for the first time in the six months ending May 15th, 1902, those who entered for the second time numbered only 483, and those who came back for a third period, 158. After this the number of returning paupers gets smaller and smaller, though solitary individuals come back for the ninth, tenth, and eleventh time. At the City poorhouse the first entries are more numerous, 3,834, but only 279 came back a second time, and 24 a third, while only 4 returned for a fourth stay, and a single person asked admission for the fifth time. This does not look as if there were, as some people declare, a large number of the poor who looked upon the poorhouse as their regular winter residence and went back again and again, while the figures also seem to indicate that the hospitality of the Barnhill establishment was more appreciated than that of the city poorhouse. It is not easy to say if a similar inference is to be drawn from the fact that, though the admissions are fewer, a larger number of the Barnhill paupers are dismissed from the poorhouse within a week of their admission—1,223 cases with 62 dependants out of 3,639 admissions, as against 874 cases with 121 dependants out of 4,142 admissions at the city poorhouse. The stay of paupers in this poorhouse is throughout longer than in the other, and if we add that in it there are a far larger number of deserted wives and children, and that the births in the poorhouse, legitimate as well as illegitimate, are more numerous, we cannot help believing that the poverty in the City district is deeper and more permanent than that which is relieved at the Barnhill poorhouse.

An interesting record is that of the causes which led to the first applications. Ill-health seems to be the chief cause of pauperism. The cases due to this are 607, but we presume that this includes not only the actual invalids—and not always these—but also their dependants. Drink is set down as the cause of poverty with 371 men and 54 females. Desertion accounts for 278 cases, improvidence for 199, and immorality for 129. In six months 180 widows and 40 orphans asked for relief. Of old people who had relatives able to assist—and why should such ever need to ask parochial relief?—there were 143, and of old people who had no such kin 127. Criminality accounts for 103, and accident for 46. Regarding the number of the aged poor who are driven to the parish for subsistence, Mr. Motion makes some trenchant remarks:—

Having noticed frequently in discussions and conferences that it was assumed that the paupers chargeable over 65 years at a given date had just become chargeable at that age, I made an examination of the figures pertaining to this parish and the following is the result: Number of paupers chargeable to the parish of Glasgow at May 15th, 1901, who were upwards of 65 years of age, 2,242; of whom the number who were under 65 when first chargeable to the parish, 1,252, or 55·84 per cent.; the number of those who had attained the age of 65 or upwards before becoming chargeable to the parish, 990, or 44·11 per cent.

These figures show that more than half of our paupers belong to a class which, as Mr. Motion says, all the old-age pensions in the world will never touch.

He quotes some cases. One man, aged 35 years and single, who had been earning 37s. 3d. a week, had to ask for parish relief after only one week's illness. A married woman of 30 asked relief, her husband, a painter, aged 32, being in hospital with small-pox. Six children had been born to this couple in 10 years. The husband had been off work for two weeks, and till then had been earning 9d. an hour (equal to about £2 a week). He did not belong to any Friendly Society. Several cases very similar to this are quoted. Even membership of a society does not always imply all it might in the way of providence. Here is a note in point:—

"I have perused a funeral undertaker's account of £8 9s. for the burial of a man who had been on our roll at 8s. a week. He had been in a Friendly Society, and his widow received about £16 14s. The account shows: Coffin, £4 4s, ; hearse, 30s.; two coaches, 26s.; while ground, only 12s. 6d.—common ground. The widow is on the roll at 24s. a month."

A new feature of the Glasgow report is a list of the applicants for medical relief during four weeks, who had not been so long as 14 days resident in Glasgow before they applied. Some had come straight from outlying towns, seeking, evidently, a favourite Poor-law infirmary as some people do a favourite hospital, and of the rest the majority had been only one day in the city, and very few as much as a week. This is, as Mr. Motion says, a form of Poor-law work which is advancing "by leaps and bounds," and may need severe restrictive measures to check it.

A useful feature of the New Monkland report is the list of outdoor paupers for the last three years, the number of dependants belonging to each, and the sums they receive weekly. This shows at a glance the increase or decrease of numbers among these paupers; which are new cases and which are old. We note with satisfaction that on the whole the numbers are decreasing, and that of those receiving relief, the majority seem to be widows with children, to whom it is not unreasonable to give some out-door help. Certainly the numbers of dependants and the amount given do not seem to bear any fixed relation. Why, for example, should Mary Reynolds, with five dependants, receive 5s. a week, and Jane Keenan, with the same number, 8s.? William Carrick, with one dependent, receives 4s., and the same sum must serve Mary Colvert and her four dependants. There may, no doubt, be a good reason for this difference of treatment, but a New Monkland ratepayer might well ask the reason for it to be stated.

The chief characteristic of the St. Peter Port account is the report of the diet-sheet of the paupers in the poorhouse, or, as it is called, the hospital, there. The food for able-bodied inmates, for invalids, and for children is shown, and seems to be very reasonable, and, which is worthy of mention, sufficiently varied. Indeed, the fare at St. Peter Port seems to indicate that kindly feeling towards those who have come under the Poor-law which is more apt to mark small communities, where the ratepayers have some possibility of knowing the circumstances of those whom they support, and where the rates are not likely to be too onerous. So interesting and suggestive are all the reports that one cannot but feel a regret in studying them, that so few of the ratepayers of the different parishes will take the trouble to read them. They pay their rates and perhaps grumble at the amount of them, but they take little trouble to see how they are spent; and this indifference makes for pauperism, because it keeps those who might do so from trying to devise other schemes for dealing with the deserving poor, and leaving the rest to a severer discipline.

